# Transvaginal cervical cerclage: double monofilament modified Wurm *vs* single braided McDonald technique

**DOI:** 10.1002/uog.29184

**Published:** 2025-02-25

**Authors:** V. Donadono, P. Koutikwar, A. Banerjee, M. Ivan, C. S. Colley, M. Sciacca, D. Casagrandi, A. Tetteh, N. Greenwold, L. M. Kindinger, K. Maksym, A. L. David, R. Napolitano

**Affiliations:** ^1^ Fetal Medicine Unit University College London Hospitals NHS Foundation Trust London UK; ^2^ Elizabeth Garrett Anderson Wing, Institute for Women's Health University College London London UK; ^3^ National Institute for Health and Care Research University College London Hospitals Biomedical Research Centre London UK

**Keywords:** cervical cerclage, cervical length, McDonald cerclage, preterm delivery, Wurm cerclage

## Abstract

**Objective:**

To compare pregnancy outcome in women at high risk of preterm birth undergoing the modified Wurm (two monofilament sutures) *vs* those undergoing the McDonald (single braided suture) transvaginal cervical cerclage technique.

**Methods:**

This was a single‐center prospective observational study of all women with a singleton pregnancy attending a prematurity surveillance clinic because of an increased risk of preterm birth, and undergoing history‐ or ultrasound‐indicated transvaginal cervical cerclage. Two cerclage techniques were evaluated and the choice of cerclage was at the physician's discretion. In the modified Wurm technique using monofilament material, two circumferential sutures are placed with two insertions each (four in total). Outcomes were compared *vs* those of women undergoing the McDonald technique (single braided suture using a diamond‐type insertion method with four insertions in total). Primary outcome was the rate of preterm birth at < 32 weeks' gestation, with planned subanalyses according to cervical cerclage indication (history‐ or ultrasound‐indicated), preterm birth rate at any gestational age (< 37, < 34, < 28 and < 24 weeks), and sonographic cervical length (CL) of ≤ 25 mm and ≤ 15 mm. Secondary outcome measures included maternal and neonatal adverse events and outcomes, including the pre‐ and postsurgical characteristics. In addition, a reproducibility analysis using Bland–Altman plots was performed to evaluate the intra‐ and interobserver reproducibility in assessment of CL on ultrasound examination before and after cerclage.

**Results:**

In total, 147 patients were included in the final analysis: 55 (37%) received modified Wurm cerclage and 92 (63%) received McDonald cerclage. Other than race, demographic characteristics were comparable between the two groups. Of these, 22 (40%) women in the modified Wurm group had history‐indicated cerclage, *vs* 50 (54%) women in the McDonald group; the remaining cerclages were ultrasound‐indicated. In women with a short CL (≤ 25 mm), there was a significantly lower rate of preterm birth at < 32 weeks' gestation after modified Wurm compared with the McDonald technique (3 (9%) *vs* 14 (29%); adjusted odds ratio (aOR), 0.25 (95% CI, 0.06–0.95); *P* = 0.042). However, the study was underpowered to provide definitive conclusions. In the overall population, there was no significant difference in preterm birth rate for < 32 weeks' gestation between the two techniques (7 (13%) *vs* 22 (24%); aOR, 0.51 (95% CI, 0.20–1.33); *P* = 0.169). There was no difference in overall surgical complications between the two techniques. The pregnancy loss rate and composite neonatal morbidity/mortality rate were comparable between the two groups (2 (4%) *vs* 7 (8%); odds ratio (OR), 0.47 (95% CI, 0.09–2.33); *P* = 0.485; and 5 (9%) *vs* 11 (13%); OR, 0.68; (95% CI, 0.22–2.09); *P* = 0.593, respectively).

**Conclusions:**

In high‐risk women with a sonographic short CL, placement of a modified Wurm cervical cerclage is associated with a lower rate of preterm birth < 32 weeks compared with McDonald cervical cerclage. Further research in larger cohorts is needed to confirm this finding and to determine if this technique reduces the preterm birth rate after elective cervical cerclage without CL shortening. © 2025 The Author(s). *Ultrasound in Obstetrics & Gynecology* published by John Wiley & Sons Ltd on behalf of International Society of Ultrasound in Obstetrics and Gynecology.

## INTRODUCTION

An estimated 13.4 million babies were born preterm worldwide in 2020 (9.9% of all births) with no rate changes in the last decade^1^. Antenatal treatments include low‐dose aspirin, vaginal progesterone, education on smoking cessation and treatment of asymptomatic bacteriuria and sexually transmitted infections^2^. In high‐income countries, cervical cerclage reduces the preterm birth rate in women with three or more previous spontaneous preterm births or late miscarriages, or with a single spontaneous preterm birth/late miscarriage and a short cervix^3^, although randomized trials have failed to demonstrate changes in perinatal mortality and severe morbidity^4^.

Different techniques for transvaginal cervical cerclage include the McDonald and Shirodkar cerclage (requiring bladder dissection), and the lesser known Wurm procedure^5^, ^6^, ^7^, ^8^, ^9^. The latter involves the placement of two sutures perpendicular to each other and, in the first paper it was described that the cervical canal is invaded. The McDonald technique involves the placement of a single braided (or less frequently monofilament) suture with a diamond insertion at four points on the cervix. There is no consensus on the best surgical method for cerclage insertion, which suture material to use or how many sutures to place. The use of monofilament suture material seems to have a lower impact on the vaginal microbiome^10^; however, a multicenter trial did not show any difference in outcome comparing monofilament *vs* braided suture material despite differences in rates of maternal infection^11^. The trial included a variety of different surgical methods and number of sutures placed, which could have affected the outcome. Retrospective observational studies have indicated that the higher the cervical cerclage is (i.e. closer to the internal cervical os), the lower is the risk of preterm delivery^12^, ^13^. In women with a failed transvaginal cerclage and spontaneous preterm birth or preterm prelabor rupture of the membranes (PPROM) before 28 weeks' gestation, the placement of a transabdominal cervical cerclage, which achieves the highest placed insertion, is more effective compared with transvaginal cervical cerclage^14^. Hence, surgical technique and the ability to place a higher cerclage may improve outcome.

We hypothesized that the modified Wurm technique may be more effective in reducing preterm birth in women with indicated cervical cerclage due to history or sonographic short cervical length (CL), compared with the McDonald technique. The aim of this study was to provide feasibility data for a larger prospective study to determine any clinically significant differences in transvaginal cerclage techniques and spontaneous preterm birth, rather than to answer a definitive question on which is the most effective technique.

## METHODS

This was a single‐center prospective observational study of all women with a singleton pregnancy undergoing history‐ or ultrasound‐indicated transvaginal cervical cerclage at University College London Hospital, London, UK, between March 2017 and December 2019. All women underwent cerclage before 24 weeks' gestation.

Exclusion criteria were: multiple gestation, rescue cerclage with exposed membranes, Shirodkar cerclage technique, McDonald technique with monofilament suture, transabdominal cervical cerclage, fetus with chromosomal abnormality or structural anomaly, and women who had iatrogenic preterm delivery.

Women included in the study underwent either elective history‐indicated cerclage or ultrasound‐indicated cerclage when the CL on transvaginal ultrasound examination was ≤ 25 mm. Women had CL monitored from 14 to 16 weeks' gestation, at a minimum interval of every 2 weeks, according to national and international guidelines, specifically if they had a previous spontaneous preterm birth ≤ 34 weeks' gestation or spontaneous late miscarriage, risk factors for preterm birth or incidental finding of a short cervix ≤ 25 mm in the current pregnancy^15^, ^16^, ^17^. All women underwent midstream urine culture and low and high vaginal swab culture before the cerclage placement in the prematurity surveillance clinic for women at risk of preterm birth. Infections were treated according to local microbiology guidelines, including antibiotic treatment for all cases of bacterial vaginosis and urinary tract infection.

At our hospital trust, four types of cerclage technique are performed either by surgeons with experience of more than 50 procedures or under their supervision: the McDonald, Shirodkar, modified Wurm and transabdominal techniques. The choice of cerclage technique performed depends on previous history, surgeon's preference, woman's request or randomized trial participation^11^.

**Figure 1:**
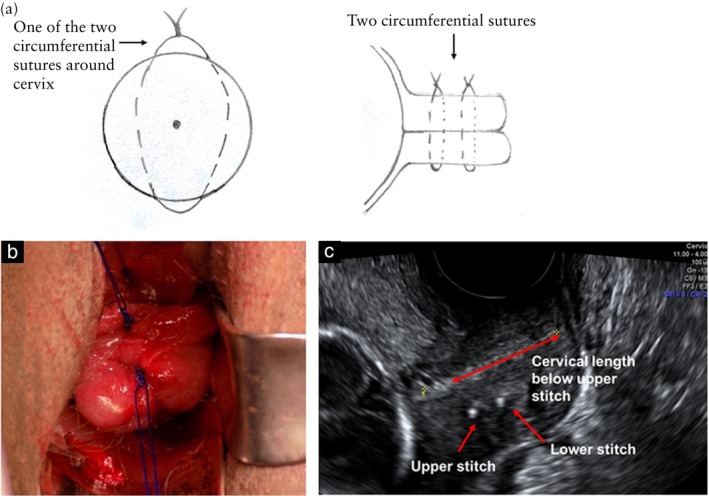
Modified Wurm transvaginal cervical cerclage technique with two monofilament sutures, each placed with two insertion points: (a) frontal (left) and lateral (right) illustration; (b) *in‐vivo* postsurgery photo; and (c) postsurgical transvaginal ultrasound image in longitudinal view.

**Figure 2:**
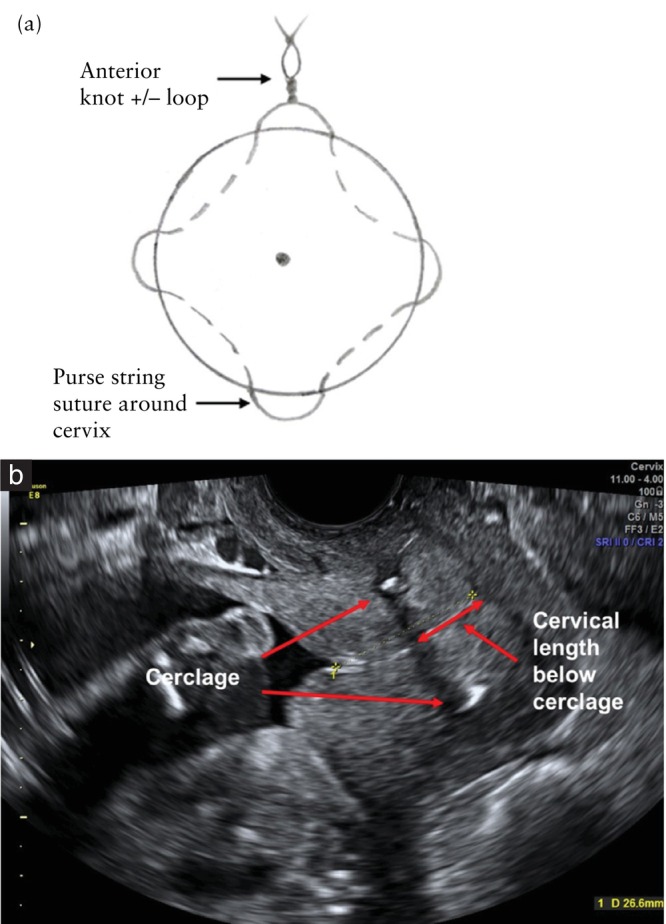
McDonald transvaginal cervical cerclage technique using single braided suture placed with four insertion points: (a) frontal illustration and (b) postsurgical transvaginal ultrasound image in longitudinal view.

The modified Wurm technique involves using monofilament material, with two circumferential sutures of which each has two insertion points in the cervix (four insertion points in total): from 12 to 6 o'clock and then from 6 to 12 o'clock with the knot tied anteriorly at 12 o'clock, without invading the cervical canal. The first suture loop is placed above the sponge holder grasping the cervical lip, and it is used as a traction to insert the second suture, which is placed as close as possible to the cervicovaginal junction (Figure 1, Videoclips S1 and S2)^7^, ^9^. The aim is to achieve a high cerclage placement of the upper suture, closer to the internal cervical os, without the need for bladder dissection. The McDonald method involves a single braided suture loop; it has four insertion points in the cervix arranged in a diamond pattern, with entry points at 12, 3, 6 and 9 o'clock (Figure 2) and with a knot tied anteriorly at 12 o'clock^5^, ^6^. The suture is placed as high as possible in the cervix.

Patients received intraoperative antibiotics prophylactically (cefadroxil 1.5 g intravenously or clindamycin 900 mg intravenously). At the time of cerclage placement some women were already on daily progesterone 200 mg vaginally until 37 weeks' gestation or they were started on it afterwards if further sonographic CL shortening was noted. All patients were followed up in the prematurity surveillance clinic every 2 weeks before and after surgery and discharged to routine or consultant‐led clinical care if no further cervical shortening or funneling through the cerclage after 24 weeks' gestation was observed. The cerclage was removed electively at 37 weeks' gestation in women aiming for vaginal delivery, unless there were signs of preterm delivery or PPROM occurred, or during elective Cesarean section at 37–40 weeks.

Maternal and neonatal records were reviewed. The primary outcome was the rate of preterm birth at < 32 weeks' gestation, with planned subanalyses according to cervical cerclage indication (history‐ or ultrasound‐indicated), preterm birth rate at any gestation (< 37, < 34, < 28 and < 24 weeks) and in women with CL of ≤ 25 mm and ≤ 15 mm. Secondary outcomes were perioperative surgical characteristics, and maternal and neonatal outcomes according to the core outcome set for evaluation of interventions to prevent preterm birth^18^. Perioperative characteristics included: gestational age at cerclage placement; CL pre‐ and postcerclage; CL above and below the highest placed suture; maternal infection status pre‐ and postcerclage; and surgical complications at cerclage placement and removal. Complications at placement of the cerclage included ruptured membranes, bladder injury, hematoma around the suture, need to repeat the cerclage, readmission for pain and persistent bleeding. Complications at removal of the cerclage were defined as retained suture material after removal attempt or need for anesthesia^11^.

Maternal outcomes included: PPROM, mode of delivery, maternal infection (defined by clinically diagnosed maternal sepsis and/or chorioamnionitis confirmed on placental histology), maternal mortality. Neonatal outcomes included: live birth, pregnancy loss (miscarriage, stillbirth, neonatal death in the 28 days after birth), termination of pregnancy, gestational age at delivery, birth weight, admission to neonatal unit, composite neonatal morbidity/mortality (defined as death, sepsis with positive culture, intraventricular hemorrhage grade > 2, periventricular leukomalacia, bronchopulmonary dysplasia, retinopathy of prematurity), congenital infection, respiratory morbidity (non‐invasive or mechanical ventilation, respiratory distress syndrome, need for oxygen therapy, chronic lung disease), gastrointestinal morbidity identified by necrotizing enterocolitis, early neurodevelopmental morbidity (defined as intraventricular hemorrhage, hypoxic‐ischemic encephalopathy, periventricular leukomalacia).

Additionally, we evaluated the reproducibility of CL measurements, both before and after cerclage placement. One operator collected the ultrasound images and measurements, while a second operator performed the measurements twice on still images to ascertain intra‐ and interobserver reproducibility of caliper placement. The repeated measurements were executed in a blinded manner using electronic calipers on archived images captured by the first operator^19^, ^20^.

The study was registered with the hospital as a Women's Health Division service evaluation project and so ethical approval was not required. Data were collected in an anonymized fashion.

### Statistical analysis

Considering a clinically meaningful reduction of severe preterm birth < 32 weeks' gestation of 10% (from 20% to 10%), with an 80% power and 0.05 alpha error, a sample of 200 women would be required per study group. For the purpose of a pragmatic feasibility study, to explore differences in preterm birth at < 32 weeks' gestation in women with short cervix (≤ 25 mm), a sample of more than 52 women per study arm (a 20% reduction from 28% to 8%), with an 80% power and 0.05 alpha error was considered adequate.

Demographic data of women who underwent modified Wurm *vs* McDonald methods were investigated by descriptive statistics. Categorical variables were presented as *n* (%). Univariate analysis for categorical variables was performed using chi‐square test or Fisher's exact test as appropriate. Parametric distribution was assessed using the Kolmogorov–Smirnov test and Shapiro–Wilk test. Continuous variables were presented as mean ± SD or median (range) and compared using Student's *t*‐test or the non‐parametric Mann–Whitney *U*‐test. A *P*‐value < 0.05 was considered statistically significant. Multiple logistic regression analysis was also conducted on the primary outcome to calculate adjusted odds ratio (aOR) and 95% CI. This analysis was not performed in the subgroup of women with CL ≤ 15 mm given the small sample size. To evaluate the agreement for the reproducibility study of CL measurement, Bland–Altman plots were used, showing the difference between paired variables against their average. The mean difference and the 95% limits of agreement (LOA) of the paired observations were calculated^21^, ^22^. Data were collected on an Excel spreadsheet (Microsoft Corp., Redmond, WA, USA) and were analyzed using the statistical package SPSS version 25.0 (IBM Corp., Armonk, NY, USA).

## RESULTS

**Figure 3:**
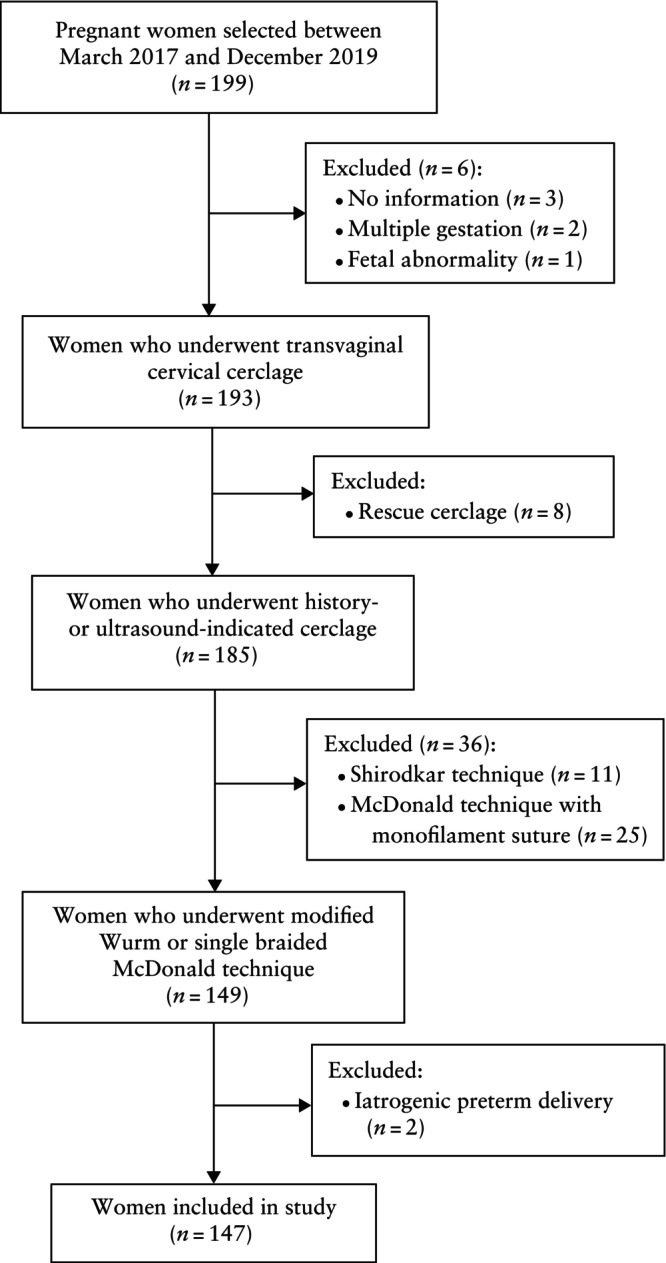
Flowchart summarizing inclusion in study of women with singleton pregnancy who underwent modified Wurm or McDonald transvaginal cervical cerclage before 24 weeks' gestation.

During the study period, 199 women underwent cerclage before 24 weeks' gestation. After excluding patients who did not meet the criteria, 147 were included in the study (Figure 3). Of these, 55 (37%) women underwent a modified Wurm cerclage and 92 (63%) had a McDonald cerclage. The characteristics of the women included are shown in Table 1. There were no differences between the two groups except for race (*P* = 0.006). In the modified Wurm group, there were more Asian and fewer black women compared with the McDonald group (31% *vs* 11% and 16% *vs* 29%, respectively).

**Table 1:** Maternal characteristics of 147 women with singleton pregnancy who underwent modified Wurm or McDonald transvaginal cervical cerclage before 24 weeks' gestation

Characteristic	Modified Wurm (*n* = 55)	McDonald (*n* = 92)	*P*
Maternal age (years)	33.6 ± 5.3	33.7 ± 4.9	0.888
Body mass index (kg/m^2^)	26.8 (18.1–46.5)	25.5 (15.8–39.2)	0.408*
Race			0.006
White	29 (52.7)	55 (59.8)	
Black	9 (16.4)	27 (29.3)	
Asian	17 (30.9)	10 (10.9)	
Smoker	0 (0)	3 (3.3)	0.293†
Gravidity	2 (1–10)	3 (1–10)	0.256*
Parity	1 (0–7)	1 (0–5)	0.496*
Prior second‐trimester miscarriage/preterm birth			
14 + 0 to 23 + 6 weeks	20 (36.4)	43 (46.7)	0.219
24 + 0 to 36 + 6 weeks	20 (36.4)	33 (35.9)	0.952
Prior PPROM	8 (14.5)	17 (18.5)	0.539
Prior cerclage	17 (30.9)	29 (31.5)	0.938
Prior FDCS	8 (14.5)	10 (10.9)	0.511
LLETZ			
≥ 1	7 (12.7)	19 (20.7)	0.223
≥ 2	3 (5.5)	5 (5.4)	1†
Cone biopsy	4/54 (7.4)	17 (18.5)	0.066
Uterine anomaly	8/54 (14.8)	6 (6.5)	0.100

Data are given as mean ± SD, median (range), *n* (%) or *n*/*N* (%).

* Mann–Whitney *U*‐test.

† Fisher's exact test. FDCS, full‐dilatation Cesarean section; LLETZ, large loop excision of transformation zone; PPROM, preterm prelabor rupture of membranes.

Indications for the cerclage were not statistically different between the two groups: 22 (40%) women in the modified Wurm group had history‐indicated cerclage, *vs* 50 (54%) women in the McDonald group (*P* = 0.092). The remaining women had ultrasound‐indicated cerclage. The two groups were not different in terms of CL, and incidence of urinary tract infection and vaginal infection before and after cerclage placement (Table 2). In terms of complications during cerclage placement, none occurred in the modified Wurm group compared with six (7%) cases in the McDonald group. Among these, three patients experienced significant pain requiring readmission, two had bleeding issues (one of whom developed a hematoma at the cerclage site followed by PPROM and miscarriage at 19 + 6 weeks' gestation) and in one patient the knot loosened and despite subsequent tightening the patient had a miscarriage at 22 + 2 weeks' gestation. There was a significantly higher number of complications at removal in seven (13%) women in the modified Wurm group compared with one (1%) woman in the McDonald group (*P* = 0.001). In the modified Wurm group, three patients required anesthesia for removal and four had partially retained sutures, whereas in the McDonald group, there was only one case of partially retained suture. In the modified Wurm group, the surgeon had difficulty in removing the suture material below the knot and embedded material was left within the cervix. In the McDonald group, partial suture material was embedded within scar tissue of the cervix and could not be removed.

**Table 2:** Perioperative characteristics in 147 women with singleton pregnancy who underwent modified Wurm or McDonald transvaginal cervical cerclage before 24 weeks' gestation

Characteristic	Modified Wurm (*n* = 55)	McDonald (*n* = 92)	*P*
Cerclage indication			0.092
History	22 (40.0)	50 (54.3)	
Ultrasound	33 (60.0)	42 (45.7)	
Prior second‐trimester miscarriage/preterm birth	16/33 (48.5)	19/42 (45.2)	0.780
Cervical length			
≤ 15 mm	10 (18.2)	9 (9.8)	0.14
≤ 25 mm	35 (63.6)	48 (52.2)	0.18
Gestational age at cerclage (weeks)	15.3 (12.1–23.7)	16.5 (12.0–23.9)	0.632*
Precerclage sludge	2 (3.6)	3 (3.3)	1†
Precerclage funneling	13 (23.6)	13 (14.1)	0.144
Cervical length (mm)			
Precerclage	23.5 ± 7.8	24.7 ± 7.4	0.319
Postcerclage	32.1 ± 6.5	31.2 ± 8.0	0.503
Below highest placed suture	17.3 ± 4.0	16.5 ± 5.3	0.259
Above highest placed suture	14.6 ± 6.4	14.7 ± 6.8	0.883
Increase between pre‐ and postcerclage	8.4 ± 7.0	6.5 ± 7.4	0.150
Vaginal progesterone	33 (60.0)	55 (59.8)	0.874
Infection			
Precerclage			
Any	22 (40.0)	52 (56.5)	0.053
Urinary tract	13 (23.6)	34 (37.0)	0.094
Vaginal	13 (23.6)	25 (27.2)	0.635
Postcerclage			
Any	29 (52.7)	60 (65.2)	0.134
Urinary tract	26 (47.3)	50 (54.3)	0.406
Vaginal	10 (18.2)	24 (26.1)	0.323
Cerclage complication			
Any	7 (12.7)	7 (7.6)	0.306
At placement	0 (0)	6 (6.5)	0.084†
At removal	7 (12.7)	1 (1.1)	0.001†

Data are given as *n* (%), *n*/*N* (%), median (range) or mean ± SD.

* Mann–Whitney *U*‐test.

† Fisher's exact test.

**Figure 4:**
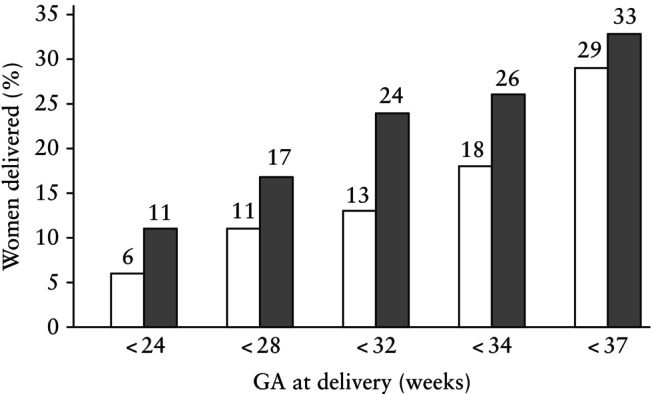
Prevalence of preterm birth at different gestational ages (GA) in 147 women with singleton pregnancy who underwent modified Wurm (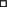) or McDonald (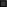) transvaginal cervical cerclage before 24 weeks' gestation.

When analyzing the primary outcome in the whole population, there was no significant difference in the rate of preterm birth at < 32 weeks' gestation between the two techniques (13% for modified Wurm *vs* 24% for McDonald cerclage; aOR, 0.51 (95% CI, 0.20–1.33); *P* = 0.169). The rate of preterm delivery < 37 weeks' gestation was not significantly different between the two groups after adjusting for race (29% *vs* 33%; aOR, 0.99 (95% CI, 0.46–2.12); *P* = 0.980). This was true for preterm delivery at any gestational age (< 34, < 32, < 28 and < 24 weeks) (Figure 4). In women with CL ≤ 25 mm there was a significant reduction in delivery < 32 weeks in women who underwent modified Wurm cerclage compared with McDonald cerclage (9% *vs* 29%; aOR, 0.25 (95% CI, 0.06–0.95); *P* = 0.042) (Table 3).

**Table 3:** Gestational age (GA) at delivery in 147 women with singleton pregnancy who underwent modified Wurm or McDonald transvaginal cervical cerclage before 24 weeks' gestation, overall and according to cervical length

GA at delivery	Modified Wurm	McDonald	*P*	OR (95% CI)	aOR (95% CI)*	*P*
Cervical length ≤ 25 mm	35	48				
< 37 weeks	9 (25.7)	16 (33.3)	0.455	0.69 (0.26–1.82)	0.77 (0.29–2.07)	0.601
< 34 weeks	5 (14.3)	14 (29.2)	0.111	0.41 (0.13–1.26)	0.44 (0.14–1.37)	0.155
< 32 weeks	3 (8.6)	14 (29.2)	0.022	0.22 (0.60–0.87)	0.25 (0.06–0.95)	0.042
< 28 weeks	3 (8.6)	10 (20.8)	0.129	0.36 (0.09–1.41)	0.38 (0.09–1.51)	0.168
< 24 weeks	2 (5.7)	5 (10.4)	0.693†	0.52 (0.10–2.86)	0.54 (0.10–3.03)	0.483
Cervical length ≤ 15 mm	10	9				
< 37 weeks	4 (40.0)	5 (55.6)	0.656†	0.53 (0.09–3.31)	—	—
< 34 weeks	3 (30.0)	5 (55.6)	0.370†	0.34 (0.05–2.26)	—	—
< 32 weeks	2 (20.0)	5 (55.6)	0.170†	0.20 (0.03–1.53)	—	—
< 28 weeks	2 (20.0)	4 (44.4)	0.350†	0.31 (0.04–2.38)	—	—
< 24 weeks	1 (10.0)	3 (33.3)	0.303†	0.22 (0.02–2.67)	—	—
All population	55	92				
< 37 weeks	16 (29.1)	30 (32.6)	0.656	0.85 (0.41–1.75)	0.99 (0.46–2.12)	0.980
< 34 weeks	10 (18.2)	24 (26.1)	0.271	0.63 (0.28–1.44)	0.67 (0.28–1.57)	0.354
< 32 weeks	7 (12.7)	22 (23.9)	0.099	0.46 (0.18–1.17)	0.51 (0.20–1.33)	0.169
< 28 weeks	6 (10.9)	16 (17.4)	0.286	0.58 (0.21–1.59)	0.63 (0.22–1.77)	0.376
< 24 weeks	3 (5.5)	10 (10.9)	0.372	0.47 (0.12–1.80)	0.55 (0.40–2.14)	0.387

Data are given as *n* or *n* (%), unless stated otherwise.

* Adjusted for race.

† Fisher's exact test. aOR, adjusted odds ratio; OR, odds ratio.

There was no significant difference in CL or CL increase postcerclage, and no difference in CL above and below the highest placed suture.

There was no significant difference in maternal and neonatal outcomes between the two groups (Table 4). In the modified Wurm group, there was one neonatal death at 5 days after birth of a baby delivered at 23 weeks' gestation, one miscarriage at 19 + 5 weeks and one termination of pregnancy following PPROM and chorioamnionitis at 22 weeks. No neonatal deaths occurred in the McDonald group, but there were seven (8%) miscarriages at a gestational age ranging between 15 + 3 and 22 + 2 weeks. There was a similar rate of pregnancy loss (4% *vs* 8%; OR, 0.47 (95% CI, 0.09–2.33); *P* = 0.485) and composite neonatal morbidity/mortality (9% *vs* 13%; OR, 0.68 (95% CI, 0.22–2.09); *P* = 0.593) in the two groups.

**Table 4:** Neonatal and maternal outcomes in 147 women with singleton pregnancy who underwent modified Wurm or McDonald transvaginal cervical cerclage before 24 weeks' gestation

Outcome	Modified Wurm (*n* = 55)	McDonald (*n* = 92)	OR (95% CI)	*P*	
Live birth	53 (96.4)	85 (92.4)	0.49 (0.09–2.29)	0.484†	
Pregnancy loss*	2/54 (3.7)	7 (7.6)	0.47 (0.09–2.33)	0.485†	
Pregnancy loss including TOP	3 (5.5)	7 (7.6)	0.70 (0.17–2.83)	0.744†	
GA at delivery (weeks)					
All population	38.0 (19.7–41.0)	37.9 (15.4–41.3)		0.487‡	
Live births ≥ 24 weeks	38.1 (25.9–41.0)	38.1 (24–41.3)		0.873‡	
Birth weight (g)	3068.5 (541.0–4210.0)	3075.0 (524.0–4200.0)		0.873‡	
PPROM	8 (14.5)	16/89 (18.0)	0.78 (0.31–1.96)	0.591	
Neonatal unit admission	15/52 (28.8)	22/77 (28.6)	1.01 (0.47–2.21)	0.973	
Composite neonatal morbidity/mortality	5/53 (9.4)	11/83 (13.3)	0.68 (0.22–2.09)	0.593	
Congenital infection	3/52 (5.8)	8/83 (9.6)	0.57 (0.15–2.27)	0.530	
Respiratory morbidity	9/53 (17.0)	17/83 (20.5)	0.79 (0.33–1.94)	0.613	
Necrotizing enterocolitis	0 (0)	1/83 (1.2)	—	1	
Early neurodevelopmental morbidity	1/53 (1.9)	5/83 (6.0)	0.31 (0.04–2.70)	0.405	
Maternal mortality	0 (0)	0 (0)	—	—	
Maternal infection	5/53 (9.4)	13/85 (15.3)	0.58 (0.19–1.72)	0.320	
Mode of delivery				0.518	
Cesarean section	27/53 (50.9)	36/83 (43.4)	—	
Spontaneous vaginal	19/53 (35.8)	38/83 (45.8)	—	
Instrumental vaginal	7/53 (13.2)	9/83 (10.8)	—	

Data are given as *n* (%), *n*/*N* (%) or median (range), unless stated otherwise. Secondary individual postnatal outcomes were not available for all patients.

* One neonatal death and one miscarriage in modified Wurm group; seven miscarriages in McDonald group.

† Fisher's exact test.

‡ Mann–Whitney *U*‐test. GA, gestational age; OR, odds ratio; PPROM, preterm prelabor rupture of membranes; TOP, termination of pregnancy.

Intra‐ and interobserver reproducibility of CL measurements on ultrasound images before and after cerclage placement are presented in Table S1 and Figure S1. Ultrasound images of CL were available, for this secondary analysis, for 103 patients, of whom 46 (45%) underwent modified Wurm cervical cerclage and 57 (55%) received McDonald technique. The gestational age at which CL was measured, including before and after the cerclage, ranged from 11 to 26 weeks' gestation. The 95% LOA were comparable both before and after cerclage for both intra‐ and interobserver reproducibility (1.9 *vs* 2.4 mm and 3.7 *vs* 4.1 mm, respectively). Notably, when measuring CL in women who underwent the modified Wurm cerclage compared with the McDonald technique, the 95% LOA was slightly larger for interobserver reproducibility (5.1 *vs* 3.2 mm).

## DISCUSSION

The aim of this study was to investigate the impact of two transvaginal cervical cerclage techniques that differ in surgical method, material used and number of sutures placed. In women with sonographic CL ≤ 25 mm, the modified Wurm cerclage was associated with a significantly lower preterm birth rate at < 32 weeks' gestation.

Several studies have investigated different surgical methods (circumferential loops *vs* diamond placement), different number of sutures placed or different suture material; however, in many of those studies there was a mixture of any of three cerclage features. It is therefore difficult to compare a single characteristic independently from the other two^11^, ^23^, ^24^.

The most reported comparison between two different cerclage methods is the Shirodkar (with bladder dissection) technique *vs* the McDonald technique. Allegedly, the Shirodkar method is known to be a high stitch as the principle is to place the suture as close as possible to the internal cervical os in women with a particularly short cervix (< 10 mm) or a previous failed low vaginal stitch (McDonald)^25^. The closer to the internal os (higher) and the more circumferentially closer to the canal (the closer to the canal from the peripheral vaginal epithelium) the cerclage is, the better is supposed to be the outcome^14^. A recent systematic review and meta‐analysis has shown a significant reduction in preterm birth at < 37 weeks' gestation (risk ratio, 0.91 (95% CI, 0.85–0.98)), also present at 35, 34 and 32 weeks, in women undergoing Shirodkar *vs* McDonald cerclage, independently of indication for cerclage, the number of sutures placed and material used^24^. The Shirodkar method has more complications for the mother, so it should be used in selected cases. It can be argued that the aim of a modified Wurm was similar to the Shirodkar technique in order to place the higher stich as close as possible to the internal cervical os.

It has been reported in magnetic resonance imaging and pathology studies that a particularly high concentration of circular muscular fibers is present at the level of the internal cervical os, which may explain why a higher cerclage placement is more effective, reinforcing the role of such fibers^26^, ^27^. A disruption of the internal os area might explain why some women after a full dilatation Cesarean section have a high risk of preterm birth^28^, ^29^. In our cohort, we could not demonstrate a significant increase in the postcerclage CL after placement of the modified Wurm cerclage; however, we noted a trend of increased CL.

Two studies did not report any difference in outcome with the placement of one or two sutures^23^, ^30^. These studies did not stratify by different technique and materials used. Our results revealed a similar preterm birth rate in the whole population; however, we observed a statistically significant difference between the two techniques used in the rate of deliveries at < 32 weeks in women with a CL ≤ 25 mm.

The placement of a second suture might be more effective only if placed above the first one to achieve a higher cervical canal closure^13^. In observational studies on rescue cerclage cases with exposed membranes, the modified Wurm technique has been reported to prolong the pregnancy beyond 28 weeks' gestation in 58% of cases^31^.

Most studies comparing different techniques focus on the use of different suture materials, with the possible advantage that monofilament could provide lower bacterial contamination^10^, ^32^. A limitation of this study is that we did not perform routine microculture of the removed cerclage; however, there was no difference in vaginal and urinary infection rates before and after cerclage placement. The C‐STICH trial is the only randomized study comparing different suture materials and it showed no difference using monofilament or braided suture in reducing the pregnancy loss rate^11^. However, the trial reported a lower rate of maternal infection in the monofilament group compared with the braided‐suture group. Although we did not observe a difference in maternal infection rates in this study, it would be interesting to explore the outcomes of different techniques using the same suture types. Women recruited into the C‐STICH trial had similar perioperative characteristics to our population, with a 60% rate of ultrasound‐indicated cerclage and a mean CL precerclage of 23 mm. Despite these similarities, we reported a lower pregnancy loss (4% *vs* 8%) as compared with the monofilament arm of the C‐STICH study; however, the two studies cannot be compared because our cohort was smaller and not randomized. From a surgical perspective, there were no complications at cerclage placement in the modified Wurm group compared with the McDonald group (six cases (7%), of which two pregnancies ended in miscarriage). Conversely, there was a higher rate of retained suture material at the time of removal in the modified Wurm group compared with the McDonald group (7% *vs* 1%), and need for anesthesia (5% *vs* 0%), which was overall statistically significant (*P* = 0.001). On qualitative analysis, it appeared that surgeons found it more difficult to visualize the knots of monofilament sutures, it being a thinner material and more embedded into the epithelial cervical scar tissue. No cerclage removal issues were reported when the surgeon removing the cerclage was the same person who performed the placement.

When considering the primary outcome in the whole population, there was no statistically significant difference in the rate of preterm delivery at < 32 weeks' gestation between the two techniques, but we observed a significant reduction of preterm birth at < 32 weeks in the modified Wurm group in women with a CL ≤ 25 mm. Overall, there was a trend towards a lower rate of preterm birth in the modified Wurm group for all gestational‐age subgroups (Figure 4). This study was underpowered, aiming to provide preliminary data to guide the design of a future prospective trial. Although our findings suggest potential trends, they are not definitive and should be interpreted with caution.

Strengths of this study include the high reproducibility of CL measurements in reporting postsurgical findings^33^. Maternal and neonatal outcomes have been documented in accordance with a recognized set of core outcomes and collected in a short period of time so that neonatal care did not change over the time^34^, ^35^. We acknowledge a few limitations to this study, such as the small number of women included; however, from a feasibility perspective, it was adequate to explore a trial design. Given the small sample size, we could not perform a subanalysis for ultrasound‐indicated cerclage cases, however, there was no observed difference in precerclage CL between the two techniques. Another limitation relates to multiple surgeons performing the procedure, albeit with high levels of experience; however, this reflects clinical practice scenarios and therefore could be seen as a strength. In addition, practitioners may not be familiar with the placement of a modified Wurm cerclage, which could limit applicability, if a center lacks an experienced provider. Ultimately, we have not assessed long‐term outcomes.

In conclusion, the modified Wurm transvaginal cervical cerclage method was associated with significant reduction in preterm birth at < 32 weeks' gestation in women with a short cervix (≤ 25 mm). However, this study was underpowered, aiming to provide preliminary data to guide the design of a future prospective trial. Therefore, adequately powered prospective studies in larger cohorts are needed to explore further these findings.

## Supporting information


**Table S1** Intra‐ and interobserver reproducibility of cervical length (CL) measurements on ultrasound before and after transvaginal cervical cerclage placement
**Figure S1** Bland–Altman plots demonstrating intra‐ and interobserver reproducibility of cervical length (CL) measurements on ultrasound in whole population: (a) before cerclage; (b) after cerclage; (c) after modified Wurm cerclage; and (d) after McDonald cerclage.

**Videoclip S1** Surgical placement of modified Wurm transvaginal cervical cerclage.

**Videoclip S2** Transvaginal ultrasound examination of cervix after placement of modified Wurm transvaginal cervical cerclage.

## Data Availability

The data that support the findings of this study are available from the corresponding author upon reasonable request.
