# Supplementary material for: Transvaginal cervical cerclage: double monofilament modified Wurm vs single braided McDonald technique
Source: Ultrasound Obstet Gynecol. 2025 Feb 25;65(3):344–52. doi: 10.1002/uog.29184 (PMC11872348; doi:10.1002/uog.29184)

Supplementary material

**Table S1** Intra- and interobserver reproducibility of cervical length (CL) measurements on ultrasound before and after transvaginal cervical cerclage placement

|  | Mean difference, mm (%) | ±95% Limits of agreement, mm (%) |
| --- | --- | --- |
| **CL before cerclage** |  |  |
| Intraobserver | 0.2 (0.6) | 1.9 (7) |
| Interobserver | 0.1 (0.7) | 3.7 (15.3) |
| **CL after cerclage** |  |  |
| Intraobserver | 0.2 (0.7) | 2.4 (7.5) |
| Interobserver | -0.3 (-0.9) | 4.1 (12.5) |
| **CL after modified Wurm cerclage** |  |  |
| Intraobserver | 0.2 (0.7) | 2.1 (6.6) |
| Interobserver | -0.3 (-0.9) | 5.1 (15.9) |
| **CL after McDonald cerclage** |  |  |
| Intraobserver | 0.2 (0.8) | 2.6 (8.2) |
| Interobserver | -0.4 (-1.4) | 3.2 (9.4) |

CL= cervical length

**Figure S1** Bland–Altman plots demonstrating intra- and interobserver reproducibility of cervical length (CL) measurements on ultrasound in whole population: (a) before cerclage; (b) after cerclage; (c) after modified Wurm cerclage; and (d) after McDonald cerclage. Data are reported as absolute values (mm) and as percentages (%).


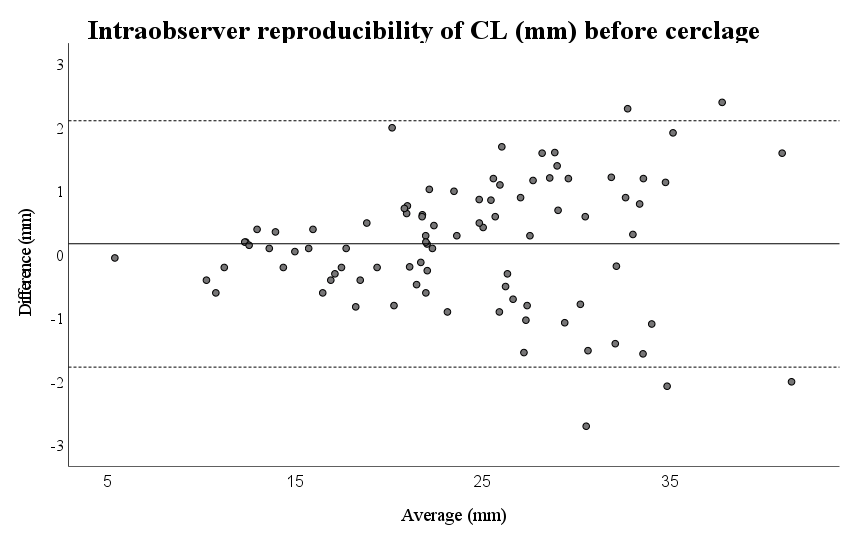

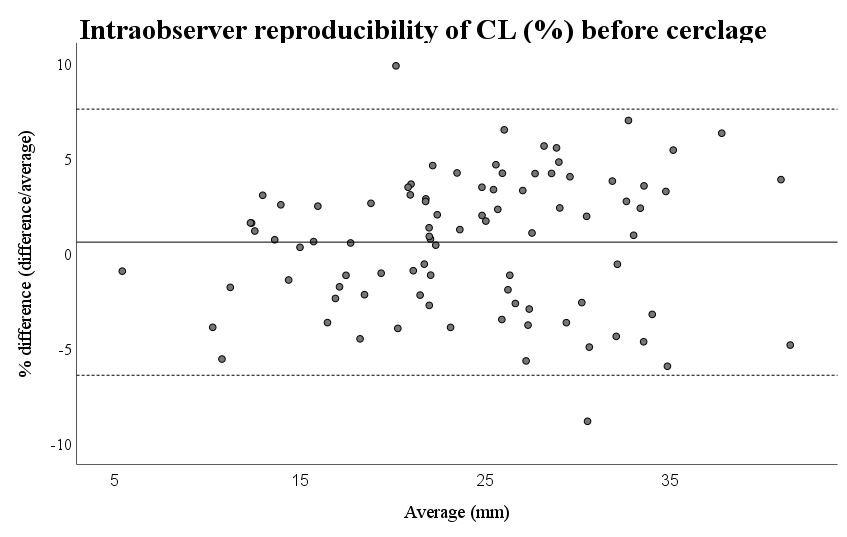


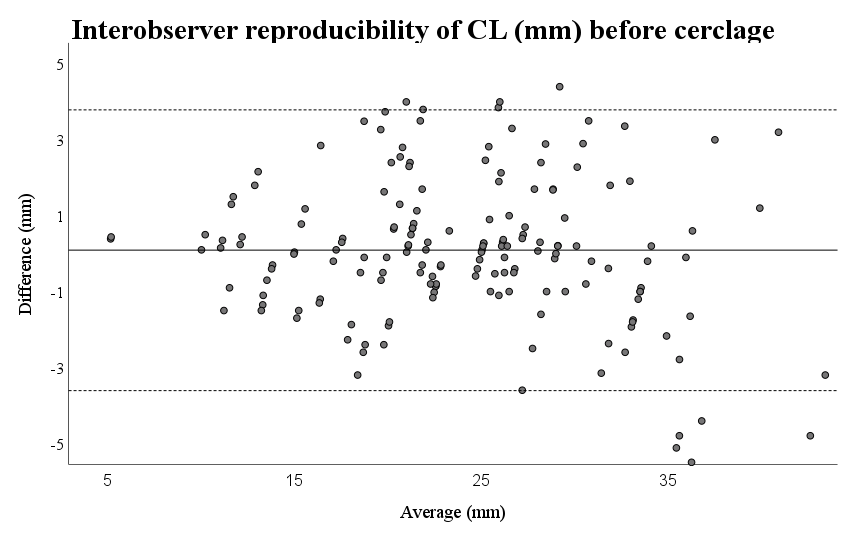

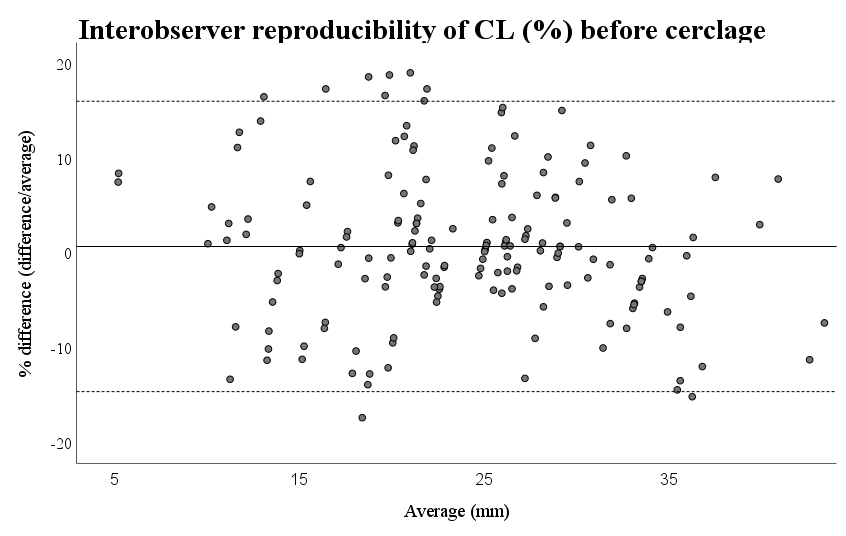


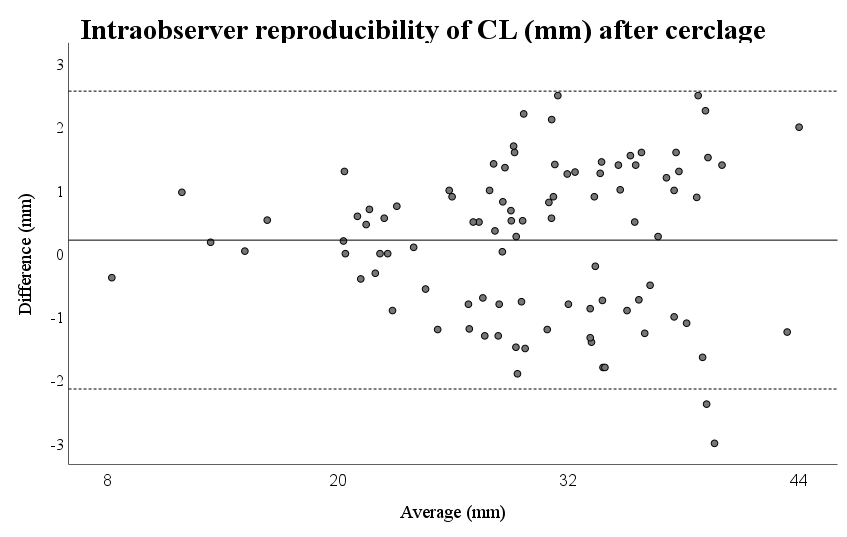

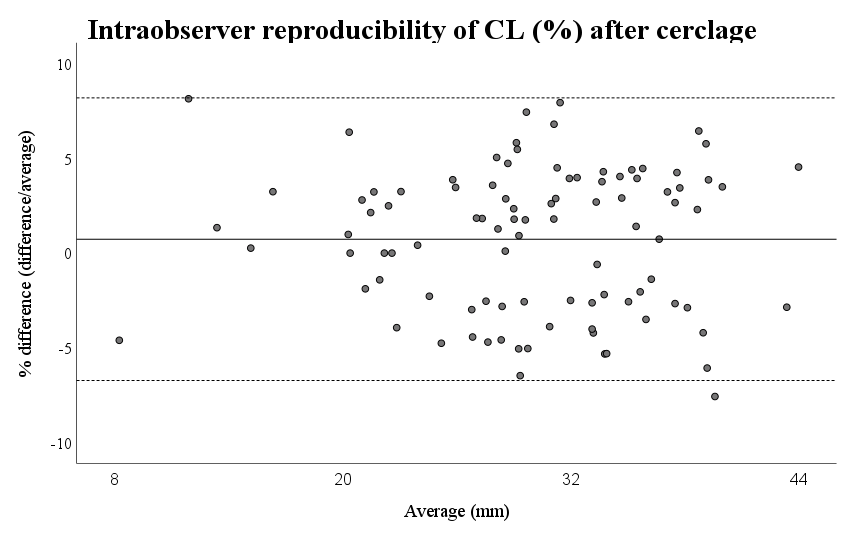


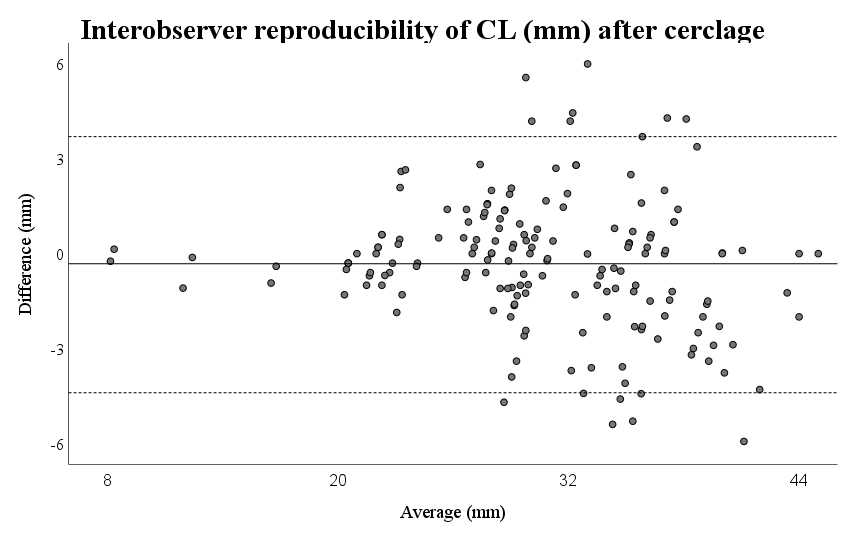

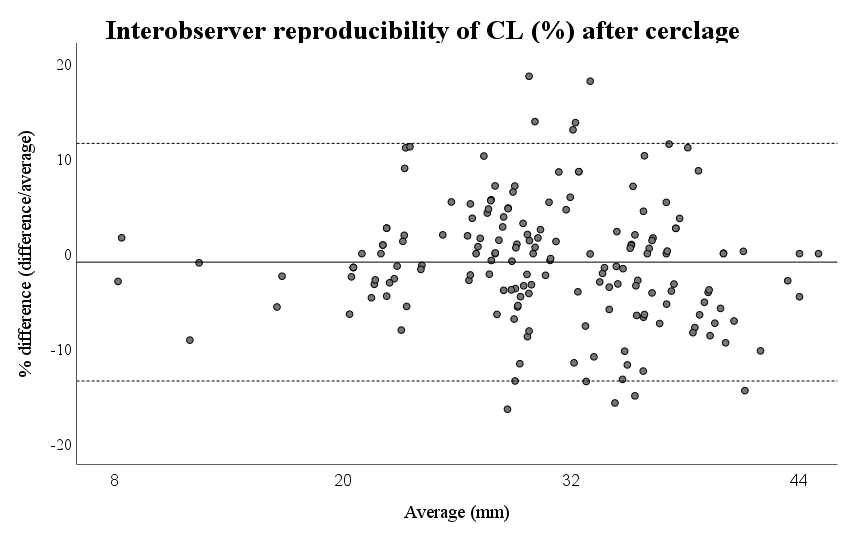


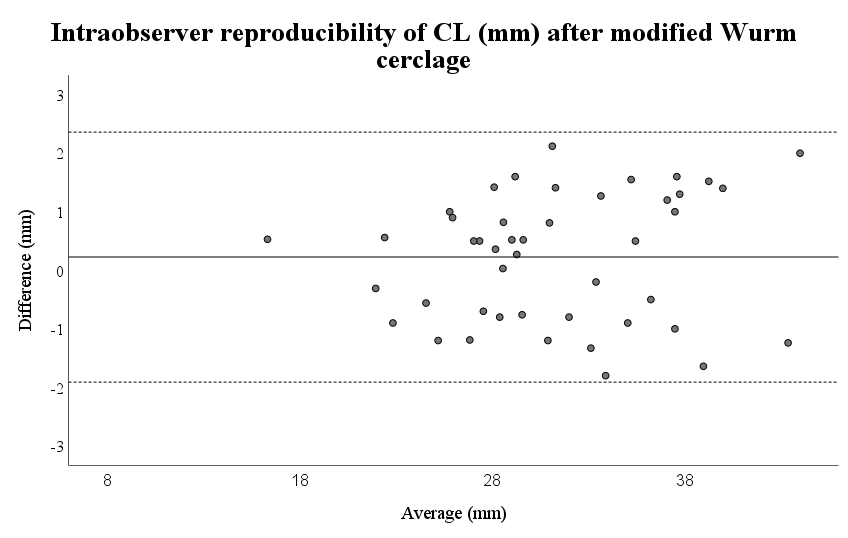

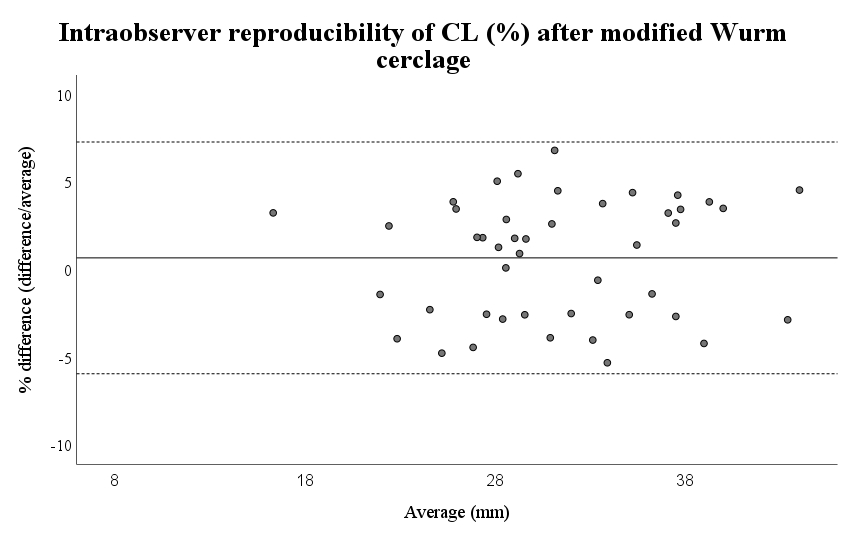


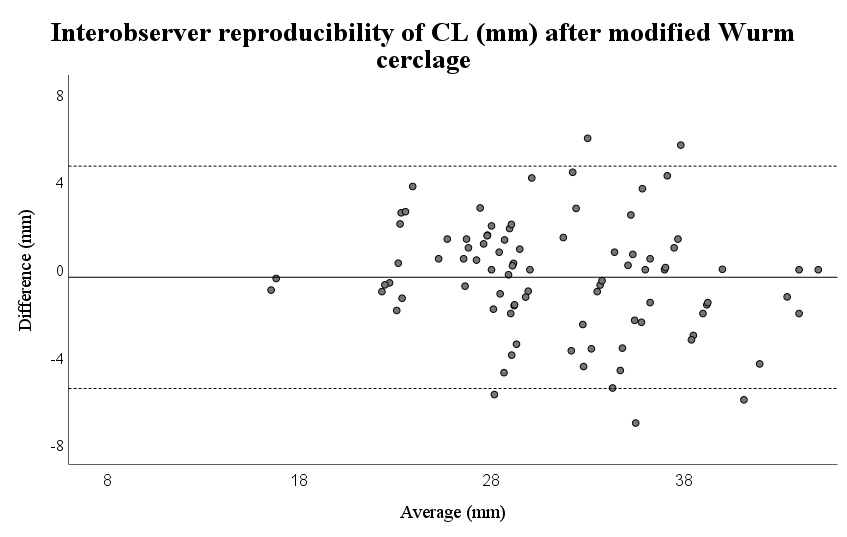

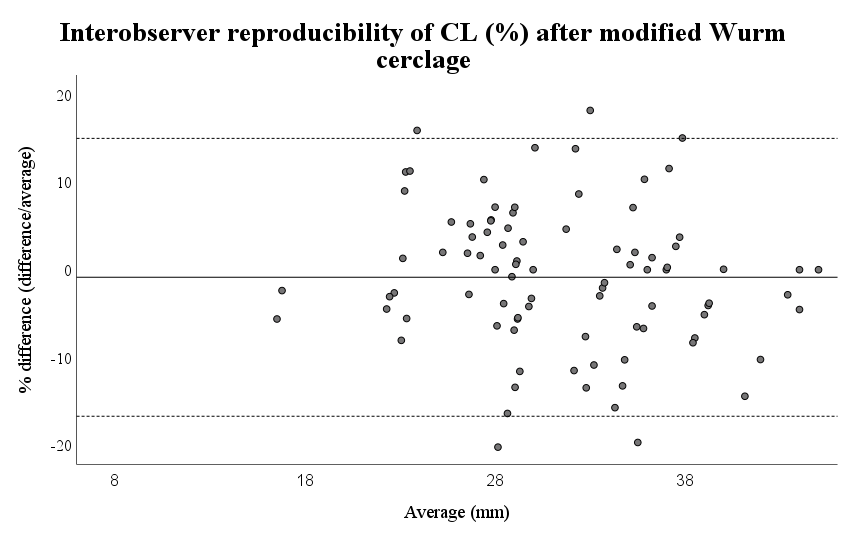


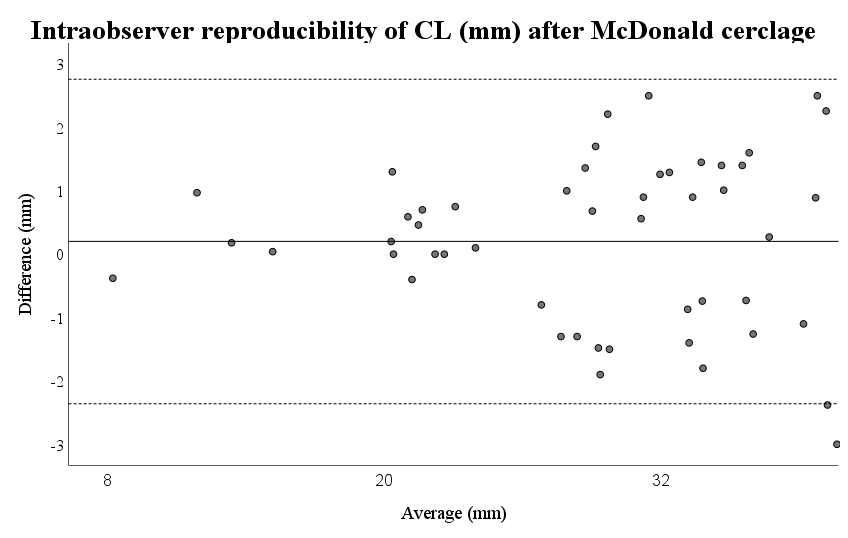

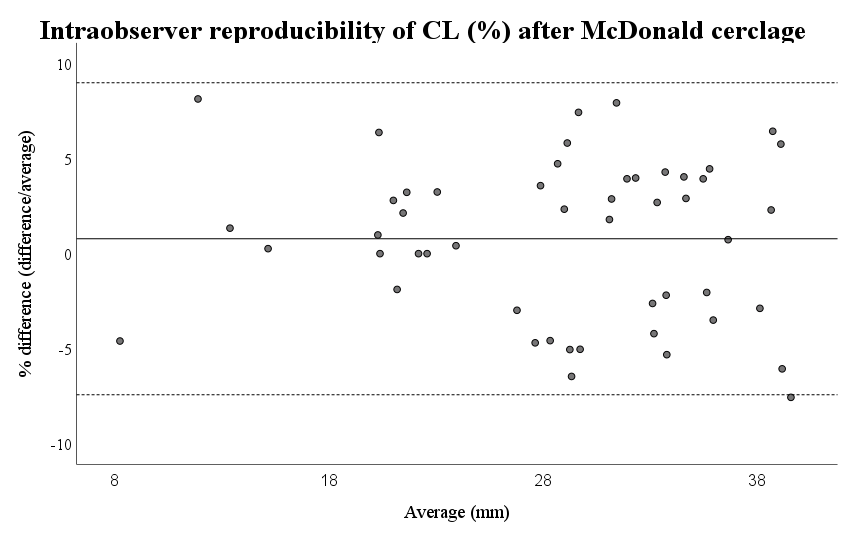


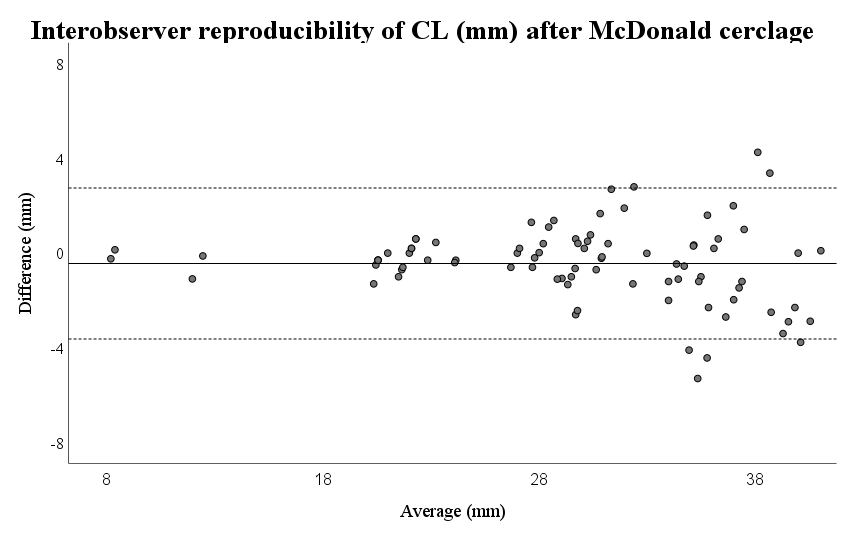

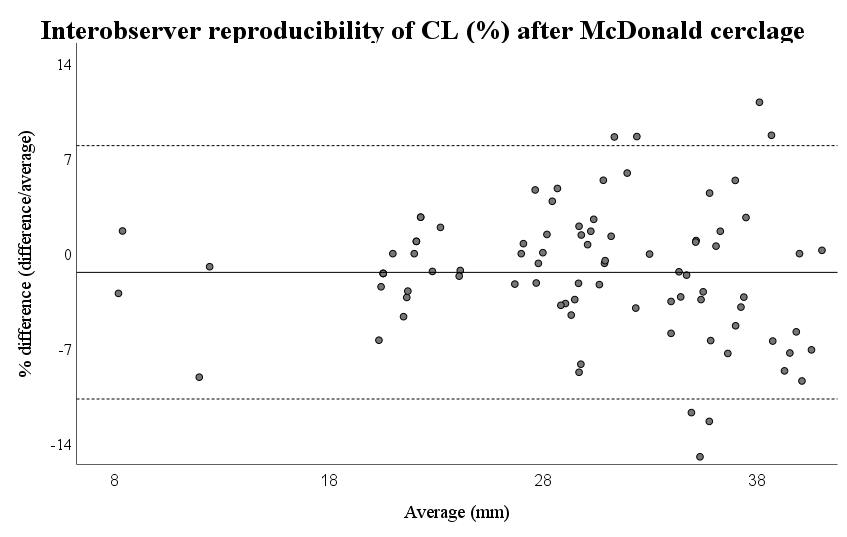

Supplement: Supplementary file 1 — Table S1 Intra‐ and interobserver reproducibility of cervical length (CL) measurements on ultrasound before and after transvaginal cervical cerclage placement Figure S1 Bland–Altman plots demonstrating intra‐ and interobserver reproducibility of cervical length (CL) measurements on ultrasound in whole population: (a) before cerclage; (b) after cerclage; (c) after modified Wurm cerclage; and (d) after McDonald cerclage. [file UOG-65-344-s001.docx]
